# Differential expression of HIF-1 in glioblastoma multiforme and anaplastic astrocytoma

**DOI:** 10.3892/ijo.2012.1555

**Published:** 2012-07-16

**Authors:** ARNULF MAYER, FABIENNE SCHNEIDER, PETER VAUPEL, CLEMENS SOMMER, HEINZ SCHMIDBERGER

**Affiliations:** 1 Departments of Radiooncology and Radiotherapy and; 2 Neuropathology, University Medical Center, D-55131 Mainz, Germany

**Keywords:** tumor hypoxia, oxygen diffusion, glioblastoma, anaplastic astrocytoma, hypoxia-inducible factor 1α

## Abstract

Hypoxia is an important factor mediating tumor progression and therapeutic resistance, in part through proteome changes mediated by the transcription factor hypoxia-inducible factor (HIF)-1. Since glioblastoma multiforme is the epitome of a highly aggressive tumor entity, while lower-grade astrocytomas often show a prolonged clinical course, a profound difference in the extent of hypoxic tissue areas and corresponding magnitude of HIF-1 activity may exist between these entities. In this study, to address this question, serial sections of 11 glioblastomas and 10 anaplastic astrocytomas were immunostained for HIF-1α, glucose transporter (GLUT)-1, carbonic anhydrase (CA) IX (i.e., hypoxia-related markers), Ki67 (proliferation), phosphorylated ribosomal protein S6 [p-rpS6; mammalian target of rapamycin (mTOR) activity] and CD34 (microvascular endothelium). Digital scans of whole tumor sections were registered to achieve geometric correspondence for subsequent morphometric operations. HIF-1α-, GLUT-1- and CA IX-positive staining was found in all 11 glioblastomas, showing a preferential expression in tissue areas adjacent to necroses. A considerable spatial overlap between GLUT-1 and CA IX, and a colocalization of these proteins with areas of enlarged mean diffusion distances were observed. Conversely, 8 of the 10 anaplastic astrocytomas were completely negative for hypoxia-related markers. The glioblastomas also showed significantly greater heterogeneity of intercapillary distances, larger diffusion-limited tissue fractions, significantly higher mTOR activity and a trend for higher proliferation rates. Microregionally, mTOR and proliferation showed a significant spatial overlap with areas of shorter mean diffusion distances. In conclusion, diffusion-limited hypoxia, leading to the expression of hypoxia-related markers is a pivotal element of the glioblastoma phenotype and may be driven by dysregulated growth and proliferation in normoxic subregions.

## Introduction

The transition from anaplastic astrocytoma (WHO grade III) to glioblastoma multiforme (WHO grade IV) is associated with a substantial increase in growth rate (1), tumor aggressiveness and resistance to therapy. While there is a meaningful proportion of patients with grade III tumors who survive for 5 years or more (2), prognosis becomes drastically poorer once the conversion to grade IV has taken place (3). For this reason, biological mechanisms behind this change in the disease course are of high interest. Hypoxia has been shown to play an important role in the malignant progression of various tumor entities (4) and, therefore, represents a candidate mechanism of this type. Both polarographic oxygen microelectrode measurements (5–10) and studies carried out with the exogenous hypoxia marker, 2-(2-nitro-1H-imidazol-1-yl)-N-(2,2,3,3,3-penta-fluoropropyl)-acetamide (EF5) (8), have shown that malignant astrocytomas do indeed contain a significant fraction of hypoxic tissue areas. Since hypoxia leads to the activation of the transcription factor hypoxia-inducible factor (HIF)-1 via stabilization of its α-subunit, the aforementioned results are supported by a number of studies which have indicated a widespread expression of HIF-1α (11–15) and its target genes glucose transporter (GLUT)-1 (16) and carbonic anhydrase (CA) IX (16,17), in malignant astrocytomas. However, most of these data do not refer exclusively to grade IV tumors, but instead suggest that hypoxic tissue areas may be prevalent even in low-grade gliomas. This is particularly true for studies using oxygen microelectrodes, which were unable to discriminate between low- and high-grade tumors. Studies of endogenous markers have in part shown a proportionality between the extent of marker expression and tumor grade (11,16,18). However, arguably, EF5 is the only surrogate marker of brain tumor hypoxia, for which a clear-cut difference between grades III and IV tumors has been identified. In a study which used both polarographic microelectrodes and EF5 to investigate the oxygenation status of grade II to IV astrocytomas (8), tumors of higher grades generally showed more abundant EF5 binding and, more importantly, quantities of EF5 corresponding to severe tumor hypoxia (approximately 0.1% oxygen) were only found in grade IV tumors.

There is currently no consensus data available regarding the question as to which of these assays can assess hypoxia most accurately. Principally, hypoxia may result from increased diffusion distances, inadequate blood flow or increased oxygen consumption rates or any combination of these factors. However, very few studies have specifically investigated these parameters in malignant astrocytomas. Among these, Rijken *et al*(19) used a glioblastoma xenograft mouse model to examine the relationship between microvessel distribution, perfusion (Hoechst 33342) and hypoxia (using the exogenous marker pimonidazole). In this experimental setting, hypoxia was caused primarily by enlarged diffusion distances, as the extent of hypoxia was found to be inversely proportional to the density of perfused microvessels. Additionally, hypoxic areas were found beyond O_2_ diffusion distances larger than 60 to 80 μm from the nearest perfused microvessels. Furthermore, Brat *et al*(20) presented data indicating that acute, perfusion-limited hypoxia may also play a role in glioblastoma, as areas of necrosis, a histological hallmark of this tumor type, often contain remnants of microvessels which show signs of intravascular thrombosis. Finally, using serial contrast-enhanced MRI and [^18^F]-fluoromisonidazole positron emission tomography (PET) studies, Szeto *et al*(21) were able to show a direct correlation between tumor proliferation and the severity of hypoxia, demonstrating the impact of increased oxygen consumption. The integration of growth factor signaling and nutrient availability is physiologically accomplished by mammalian target of rapamycin (mTOR), an evolutionary conserved serine/threonine kinase, which regulates protein translation. It has been previously demonstrated that hypoxia can inhibit mTOR activity (22), thereby avoiding the metabolic depletion of available oxygen to the point where hypoxia becomes limiting for oxidative phosphorylation (a strategy, which has been referred to as ‘oxygen conformance’) (23). In glioblastoma, this protective mechanism may at least partially be abrogated by overactive phosphoinositide-3-kinase (PI3K)-AKT signaling (24,25), which is an important upstream positive regulator of mTOR.

Additionally, a previous study established a direct link between mTOR activity and HIF-1 protein abundance (26). However, to the best of our knowledge, no study has thus far specifically addressed the relative contributions of these mechanisms and their correlations with each other in malignant astrocytomas, especially in human subjects. Therefore, the present study was carried out to address this question and, thereby, to contribute to the possible identification of the differences between the oxygenation status of anaplastic astrocytomas and glioblastomas. We investigated the expression pattern and intensity of endogenous markers of acute and chronic hypoxia (HIF-1α, GLUT-1 and CA IX), proliferation (Ki67) and mTOR status [using an antibody against the ribosomal protein S6 phosphorylated on serines 235/236 (p-rpS6) as a surrogate marker] in surgical resection specimens of 10 anaplastic astrocytomas and 11 glioblastomas. Using computer-aided image registration and analysis, the correlation of these markers with each other and with parameters derived from the abundance and location of CD34-positive tumor microvessels was analyzed.

## Materials and methods

### Samples and tumors

Histological sections of 11 glioblastomas and 10 anaplastic astrocytomas were obtained from the archive of the Department of Neuropathology, University Medical Center, Mainz, Germany. Other than tumor grade, no patient data were evaluated. The study was approved by the local medical ethics committee (Landesärztekammer Rheinland-Pfalz).

### Immunohistochemistry

Histological slides were prepared from paraffin blocks and dried overnight at 37°C. The following day, specimens were dewaxed in 2 changes of fresh xylene and then rehydrated in a descending alcohol series. The retrieval of antigenic binding sites was carried out by heating specimens in appropriate buffers (see Table I for details) in a steamer (Braun FS 10, Braun, Kronberg, Germany) for 40 min. The primary antibodies and incubation conditions used are also listed in Table I. Two different detection systems were applied: biotin-free micropolymer-based Vector ImmPRESS kits (Vector Laboratories, Burlingame, CA, USA) were used for GLUT-1, CA IX, Ki-67, CD34 and p-rpS6. HIF-1α was detected using the catalyzed signal amplification system (Dako CSA, Cat. no. K1500; Dako, Hamburg, Germany) to increase sensitivity. As the CSA system is biotin-based, endogenous biotin was blocked using prefabricated reagents from the same manufacturer (Dako, Cat. no. X0590). Preceding methodological experiments showed that endogenous biotin can lead to an unspecific staining component that is difficult to discriminate from the true antigen reactivity when this step is omitted (data not shown). In the case of the micropolymer-based procedure (Vector ImmPRESS), the reagents were applied for 90 min at room temperature. Immunodetection with the Dako CSA system (HIF-1α) was carried out following the manufacturer's instructions, except that all reagents were brought to a temperature of exactly 25°C using an Eppendorf Thermomixer compact (Eppendorf, Hamburg, Germany) before application. Negative control specimens were incubated in phosphate-buffered saline (PBS) without the primary antibody under the same conditions. Diaminobenzidine (DAB) was used as the peroxidase substrate. Slides were counterstained with Mayer's hematoxylin, dehydrated in an ascending alcohol series, and covered with a coverslip using Eukitt mounting medium (Roti-Histokitt; Carl Roth, Karlsruhe, Germany). Digital images of the specimens were acquired using a histology scanner (OpticLab H850, Plustek, Taipei, Taiwan). Additional images for illustration purposes were acquired using a microscope (AxioImager, Zeiss, Oberkochen, Germany) equipped with a digital camera (AxioCam HRc, Zeiss) connected to a Microsoft Windows-based PC running AxioVision (Zeiss), Photoshop CS4 (Adobe Systems Inc., San Jose, CA, USA) and ImageJ software (http://rsbweb.nih.gov/ij/).

### Evaluation of immunostaining

Only immunostaining compatible with the known biological function and corresponding subcellular localization of each antigen was considered as being evaluable as marker expression (Table I). Notably, faint and often diffuse cytoplasmic staining for GLUT-1 and HIF-1α, which occurred in some areas of the tumors, was not assessed. Additionally, some tumor cells showed positivity for CD34, which was not included in the calculation of microvessel-derived parameters.

### Image preparation and registration

**Figure 1:**
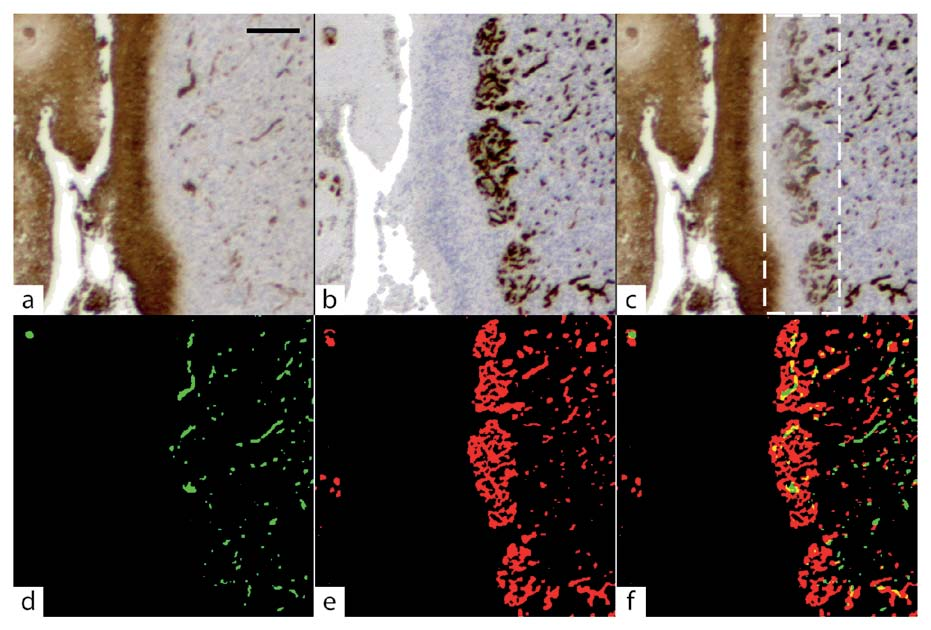
Geometric correspondence achieved by computer-aided image registration. Depicted are 400x400 pixel selections from (a) GLUT-1- and (b) CD34-stained tumor sections of a glioblastoma, taken from an area which contained characteristic microvascular proliferates in the immediate vicinity of tumor cells which exhibit strong GLUT-1 staining. The optical resolution of the scanning system (see Materials and methods) was high enough to resolve individual microvessels. The GLUT-1 specimen (a) is the source image which had been registered to the corresponding CD34 target image (b). (c) Fusion image of the left side of image (a) and the right side of image (b). In the area delineated by the white rectangle in (c), images (a and b) were blended into each other using the gradient tool in Adobe Photoshop CS4. Brown-stained vessel structures from GLUT-1 and CD34 images show plausible colocalization. GLUT-1-positive microvessels from image (a) were thresholded and are shown in green color in (d). Similarly, thresholded CD34-positive pixels from (b) are shown in red color in (e). (f) Overlay of (d and e), in which yellow pixels indicate colo calization. Both antigens coalesce to morphologically plausible structures consistent with microvessels. Notably, autochthonous GLUT-1-positive microvessels are found in the center of the CD34-positive microvascular tufts. Scale bar in the upper right corner of (a) 250 μm.

Scanned images of whole tumor sections stained for each antigen were examined simultaneously on the computer monitor to identify tumor regions which consistently contained viable tumor tissue and were present in acceptable quality for all antigens under investigation. Micronecroses within viable tumor tissue were considered acceptable, but all regions of gross necrosis were excluded from further analysis. Tumor regions discarded during this initial step were not included in any further analyses. Using different variations of the ‘lasso tool’ in Adobe Photoshop CS4, appropriate tumor areas were subsequently outlined in the CD34 image. To enable spatial correlations between different antigens and secondary parameters derived from them, digital images of scanned immunostains of HIF-1α, GLUT-1, CA IX, Ki67 and p-rpS6 were registered to the corresponding CD34 image. To achieve this, each source image (e.g., HIF-1α) and the corresponding target image (CD34) were opened simultaneously in ImageJ. Using the ‘multipoint tool’, approximately 50 to 200 corresponding landmarks were set in each image. The point selection was then converted to a ‘region of interest’ (ROI) and saved to a disk for later reference. Using the plugin ‘bunwarpj’ [http://fiji.sc/wiki/index.php/BUnwarpJ, (27)] both images were registered using the following parameters: registration mode, ‘mono’; initial deformation, ‘fine’; final deformation, ‘super fine’, landmark weight = 1, image weight = 1, verbose = yes. All other parameters were left at their default settings. A table containing statistical data regarding the registration results was saved to a disk. Using this method, registration results showed geometric correspondence down to the level of relevant microstructures (e.g., microvascular proliferates) (Fig. 1).

### Estimation of intercapillary distances (ICDs)

**Figure 2:**
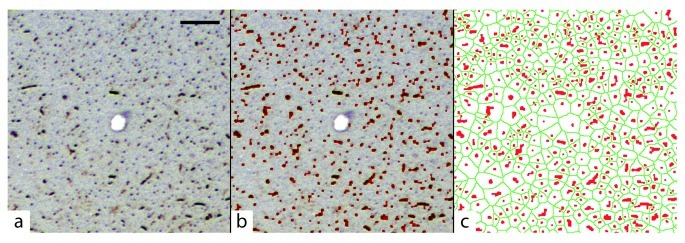
Plausibility of the Voronoi diagram. (a) A 400x400 pixel selection from a scanned CD34-stained tumor section of a highly vascularized anaplastic astrocytoma. (b) CD34-positive pixels, as identified by thresholding and subsequent binary operations (see text for details) are shown as a red overlay on the same area as depicted in (a). Microvascular domains, as defined by the Voronoi diagram (green) of CD34-positive microvessels (red), are shown in (c). Scale bar in the upper right corner of (a) 250 μm.

ICDs were measured by determining the area of the ‘microvascular domain’ surrounding each individual microvessel and deriving the diameter of a virtual circle of the same area, adapting the method of Yoshii and Sugiyama (28). The procedure involved the following steps: first, the positive pixels in the CD34 image were highlighted by color thresholding using the ‘wand tool’ in Adobe Photoshop CS4. We aimed to capture all CD34-positive pixels, but priority had to be given not to include any pixels which did not belong to CD34-positive blood vessels (e.g., debris, slight amounts of background staining), a strategy which may be referred to as ‘underthresholding’. In a second step, the selection was converted to a binary B/W mask, which was transferred to ImageJ. Due to underthresholding, these masks often contained pixels in very close proximity to each other, which - upon comparison with the original CD34 image - can be identified as being part of identical vessel structures. Different approaches were tried to correct for this error (data not shown). The following procedure turned out to be the best compromise for the tumors studied in this study and was applied as a third step: first, 3 iterations of the ‘dilate’ command were applied, which were followed by the ‘fill holes’ procedure and 2 iterations of the ‘erode’ command, resulting in a net dilation of the vessel mask of one pixel. The resulting pixel mask was overlaid onto the original CD34 image and checked for plausibility. The resulting mask typically covered the entire vessel structure (Fig. 2a and b). In a fourth step, the binary mask was used to calculate the corresponding Voronoi tessellation, which is equivalent to the microvascular domains around each separate microvessel (Fig. 2c). Since some blood vessels may occupy a considerable fraction of the area of their microvascular domain, area measurements of the latter were corrected by subtraction of the number of CD34-positive pixels from the total number of pixels in each area. Finally, the distribution of microvessel domain areas and the corresponding standard deviations were calculated in ImageJ and converted to the corresponding calibrated ICD values.

### Calculation of diffusion-limited fractions (DLFs)

To estimate the fraction of tissue experiencing chronic hypoxia, we calculated the extent of tumor area beyond a distance of 80 and 120 μm to the nearest microvessel. Haustermans *et al* published a similar study on rectal cancer (29) and referred to this measurement as the DLF. The choice of cut-off points for our study of malignant astrocytoma tissue was based on data from Rijken *et al*(19), who showed that the binding intensity of the exogenous hypoxia markers, pimonidazole and 7-(4-(2-nitroimidazol-1-yl)-butyl)-theophylline (NITP), in a human glioma xenograft model increased significantly beyond approximately 80 μm and reached their highest values beyond a diffusion distance of 100 μm. Additionally, Groebe and Vaupel (30) published calculated diffusion radii at the beginning (= arterial end) of tumor microvessels of similar magnitude. In detail, the CD34 pixel mask (see above) was used to calculate an Euclidean distance map, i.e., a gray level image of the exact same dimensions as the original CD34 image, in which the gray value of each pixel is directly proportional to its distance from the nearest microvessel within an 8-bit dynamic range. A gray value histogram of this image represents the distribution of diffusion distances in the original image. The fractions of the number of pixels above a calibrated distance of 80 and 120 μm were calculated and designated as DLF_80_ and DLF_120_, respectively.

### Quantification of marker expression

The extent of the expression of HIF-1α, GLUT-1, CA IX, Ki67 and p-rpS6 was quantified as the positive fraction of the total evaluable tumor area. As a consequence of the registration procedure outlined above, the total tumor area in individual tumors was identical for all antigens. Marker-positive pixels were highlighted by color thresholding in Adobe Photoshop CS4 using the ‘wand tool’, while simultaneously examining the corresponding glass slides under the microscope to avoid capturing regions which did not belong to cancer cells. This was especially important in the case of GLUT-1, which is not only found in cancer cells but also exhibits a physiological expression in the endothelium of autochthonous brain capillaries and the cell membrane of red blood cells (31). The resulting selection was used to generate a B/W binary mask image which was saved to a file for colocalization analysis. In an analogous manner, mask images were generated for the total evaluable tumor area. Using ImageJ, the number of positive pixels per total tumor area was calculated.

### Colocalization studies

Binary masks of HIF-1α, GLUT-1, CA IX, Ki67 and p-rpS6 generated in the previous step were used to investigate mutual colocalization patterns of these antigens. GLUT-1 was used as the reference for hypoxic tissue areas. To characterize the degree of overlap between individual markers we calculated an ‘overlap co-efficient’ (OC):

$$\text{OC}=\frac{\text{pixels}\;\text{of}\;\text{marker}\;\text{A}\;\text{colocalizing}\;\text{with}\;\text{marker}\;\text{B}/\text{pixels}\;\text{of}\;\text{marker}\;\text{B}}{\text{pixels}\;\text{of}\;\text{marker}\;\text{A}\;\text{not}\;\text{colocalizing}\;\text{with}\;\text{marker}\;\text{B}/\text{pixels}\;\text{in}\;\text{the}\;\text{entire}\;\text{tumor}\;\text{area}\;\text{negative}\;\text{for}\;\text{marker}\;\text{B}}$$

In the case of a completely equal distribution of marker A within tumor areas positive for marker B, the value of OC = 1. With increasing colocalization, the OC rises, while it falls below 1 in the case of increasing colocalization of marker A with areas negative for marker B. OC is not defined in the case of a perfect (i.e., 100%) colocalization.

### Statistical analyses

All statistical analyses were performed using the SPSS software package (version 20, IBM, Armonk, NY). The significance level was set at α = 5% for all comparisons. Linear correlations between 2 variables were described by Spearman's rank correlation coefficient (ρ). Two-sided Mann-Whitney U tests and Wilcoxon tests were used for comparison of categorized variables.

## Results

### Expression of hypoxia-related markers

**Figure 3:**
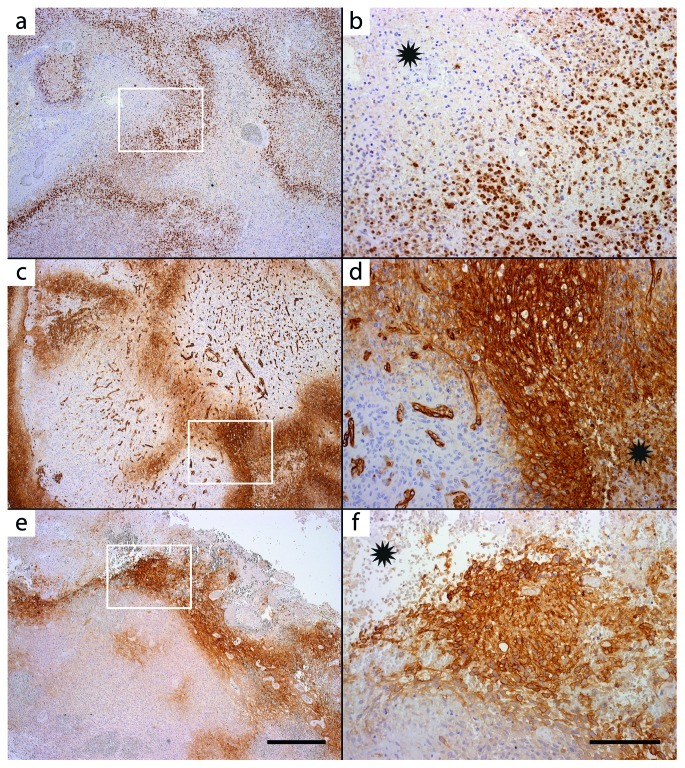
Expression of HIF-1α, GLUT-1 and CA IX in glioblastomas. Microscopic images (a, c and e), magnification, x50, scale bar in (e) 500 µm. White rectangles denote areas which are shown in magnification, x200 in (b, d and f), scale bar in (f) 150 µm. Glioblastomas showed a strong nuclear signal for HIF-1α (a and b), preferentially located in perinecrotic tumor regions [black star in (b)]. Glioblastomas also invariably showed strong membranous staining for GLUT-1 (c and d) and CA IX (e and f), which was preferentially found in tissue areas adjacent to or surrounding necrosis [black stars in (d and f)].

**Figure 4:**
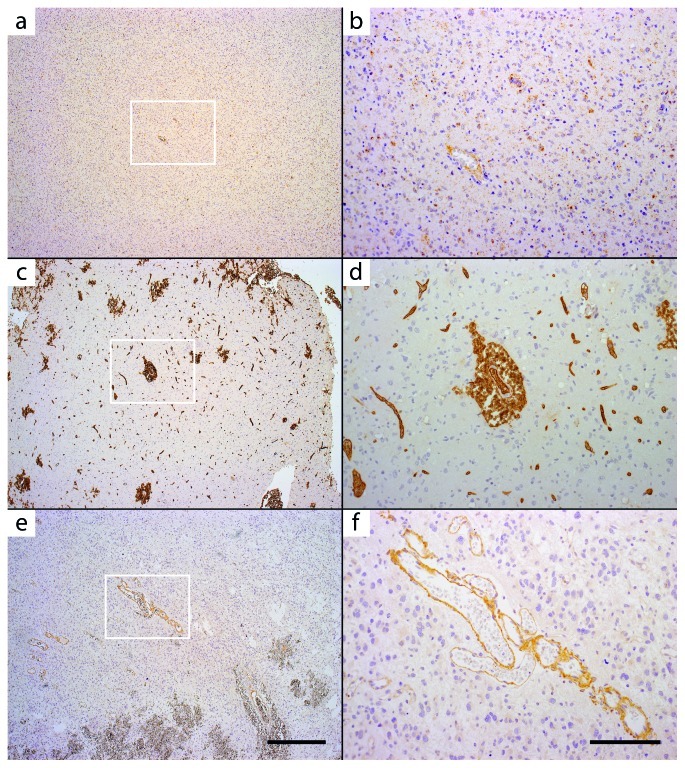
Expression of HIF-1α, GLUT-1 and CA IX in anaplastic astrocytomas. Microscopic images (a, c and e), magnification, x50, scale bar in (e) 500 µm. White rectangles denote areas which are shown in magnification, x200, in (b, d and f), scale bar in (f) 150 µm. Most anaplastic astrocytomas showed no nuclear staining of HIF-1α. Only a faint, speckled cytoplasmic signal is observed (a and b). Both GLUT-1 (c and d) and CA IX (e and f) are also almost completely absent from neoplastic cells in the majority of anaplastic astrocytomas. Expression of GLUT-1 in autochthonous blood vessels and in red blood cells (c and d) is a physiological finding and serves as an endogenous positive control for the IHC assay. Faint cytoplasmic staining for CA IX is present in a subset of microvessels (e and f).

All 11 glioblastomas were positive for all 3 hypoxia-related markers (HIF-1α, GLUT-1 and CA IX). Regions of micronecrosis were almost invariably surrounded by staining for GLUT-1 or partially surrounded by staining for the other 2 hypoxia-related markers, but staining of all 3 markers additionally occurred independent of necrosis (Fig. 3a–f). The extent of marker expression in individual tumors was highly heterogeneous. The expression of GLUT-1 was most abundant (Table II). Conversely, only 2 out of the 10 anaplastic astrocytomas showed expression of all three hypoxia-related markers, while the remaining 8 tumors were completely negative (Fig. 4a–f). The extent of GLUT-1 expression highly correlated with the expression of both HIF-1α (ρ=0.76, p<0.0001) and CA IX (ρ=0.87, p<0.0001). For all 3 markers, the difference in antigen expression between glioblastoma and anaplastic astrocytoma was highly significant (Table II).

### mTOR activity and proliferation

Similar to hypoxia-inducible proteins, intertumor heterogeneity of proliferation and mTOR activity was pronounced. mTOR activity (as assessed by the abundance of p-rpS6) was significantly higher in glioblastomas compared to anaplastic astrocytomas, while there was only a trend for higher proliferation (Ki67) in these tumors (Table II). Both markers were significantly more abundant in GLUT-1 positive tumors (mTOR: ρ=0.55, p<0.009; Ki67: ρ=0.51, p<0.017). Additionally, there was a correlation between the extent of p-rpS6 and Ki67 staining (ρ=0.59, p=0.005).

### ICD and DLF

**Figure 5:**
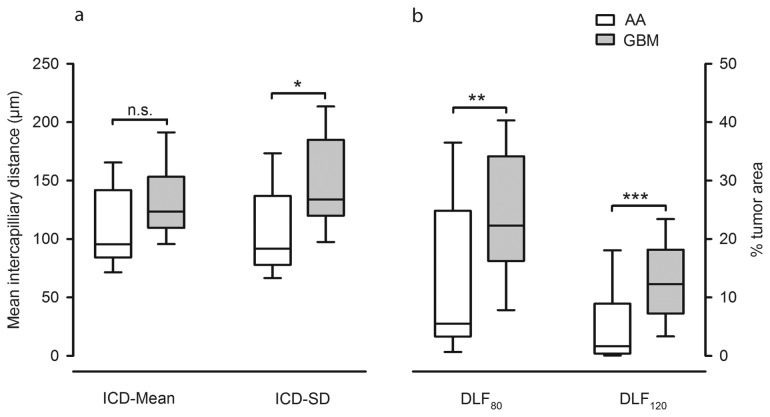
Comparison of microvessel-associated parameters in glioblastoma vs. anaplastic astrocytoma. (a) The distributions of mean values (ICD-M) and standard deviations (ICD-SD) of intercapillary distances of glioblastomas (n=11, grey) and anaplastic astrocytomas (n=10, white). Glioblastomas exhibited significantly larger standard deviations of intercapillary distances (^*^p=0.02). As shown in (b), glioblastomas exhibited a larger proportion of diffusion-limited tumor areas (^**^p=0.043; ^***^p=0.006). AA, anaplastic astrocytoma; GBM, glioblastoma multiforme; DLF, diffusion-limited fraction.

Mean ICDs were slightly larger in glioblastomas compared to anaplastic astrocytomas, although this difference was not statistically significant. However, standard deviations of ICDs were found to be significantly different between these entities, with glioblastomas showing higher values (p=0.02, Fig. 5). Importantly, compared to anaplastic astrocytomas, glioblastomas showed significantly larger areas experiencing chronic hypoxia, as assessed by the parameters DLF_80_ (p=0.043) and DLF_120_ (p=0.006). For both parameters, the respective fractions of the tumor areas were approximately twice as large in glioblastomas compared to anaplastic astrocytomas (Fig. 5). The standard deviation of ICDs, DLF_80_ and DLF_120_ also showed positive correlations with GLUT-1 (ρ=0.51, ρ=0.46 and ρ=0.58, respectively, p<0.05) and HIF-1α 1 (ρ=0.48, ρ=0.44 and ρ=0.55, respectively, p<0.05), while CA IX only correlated with DLF_120_ (ρ=0.52, p=0.016).

### Correlations between marker expression and diffusion distances

Point-to-point registration of all images to the CD34 reference image allowed the analysis of the distribution of diffusion distances in marker-positive vs. marker-negative areas. As expected, significantly higher mean diffusion distances were observed in GLUT-1 and CA IX-positive areas compared to the corresponding marker-negative areas (p=0.003 for both). There was also a trend for higher mean diffusion distances in HIF-1α positive areas, but the level of statistical significance was not reached. Areas positive for the mTOR surrogate marker, p-rpS6, and the proliferation marker, Ki67, both contained significantly shorter mean diffusion distances compared to marker-negative areas (p=0.001 and 0.006, respectively) although the absolute magnitude of this difference was modest.

### Colocalization analysis

**Figure 6:**
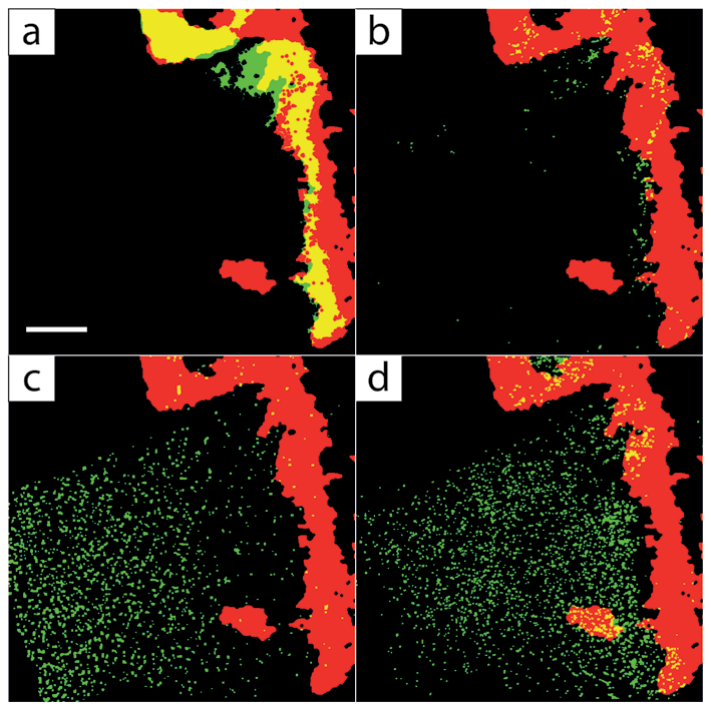
Colocalization analysis. 800x800 pixel selection from a glioblastoma showing merged images of GLUT-1 (red) and (a) CA IX, (b) HIF-1α, (c) Ki67 and (d) p-rpS6. The latter antigens are each shown in green. Yellow indicates colocalization. (a) GLUT-1 and CA IX exhibit substantial overlap. (b) HIF-1α expression is sparse and overlap is less evident. (c) Ki67 is preferentially located in GLUT-1-negative areas, while p-rpS6 shows a similar tendency but its expression is more pronounced in areas directly adjacent and even overlapping with GLUT-1-positive regions (d). Scale bar in (a) 500 μm.

In addition to the quantitative correlations described above, we also investigated the spatial colocalization of marker expression. As the aforementioned analyses had shown that GLUT-1 is the most abundant marker of chronic hypoxia, the latter was chosen as the reference marker for this purpose. The degree of colocalization between CA IX and GLUT-1 was very high, exhibiting a mean OC value of 64.1 (range, 3.1 to 340.6), (Fig. 6a). As CA IX staining was less extensive than GLUT-1 staining, regions of CA IX positivity were often found as subareas within the GLUT-1-positive areas. Only a partial colocalization was found between GLUT-1 and HIF-1α (mean OC = 5.9; range, 0 to 28.6), (Fig. 6b). Only parts of the GLUT-1-positive area were positive for HIF-1α and there were additional HIF-1α-positive regions in well-vascularized areas. Mean OC values for p-rpS6 and Ki67 were <1 (0.81 and 0.72, respectively), indicating a tendency for the expression outside of the hypoxic regions. The degree of this pattern was variable, with selected tumors showing almost a mirror-image pattern of the hypoxic regions (Fig. 6c and d). Of note, both the latter antigens displayed only a minor degree of colocalization with each other (mean OC = 1.98).

## Discussion

In this study, we employed a novel approach using computerized image analysis of multiparametric immunohistochemistry in registered serial whole tumor sections to systematically investigate the hypoxia-related pathophysiology of glioblastoma and anaplastic astrocytoma. To the best of our knowledge, this is the first study clearly showing that glioblastomas and anaplastic astrocytomas differ fundamentally as regards the expression of the hypoxia-related markers, HIF-1α, GLUT-1 and CA IX. All of the investigated glioblastomas, but only 2 anaplastic astrocytomas were positive for all 3 of these proteins. By contrast, the remaining 80% of anaplastic astrocytomas were negative for all 3 hypoxia-related markers. This finding is consistent with a recent meta-analysis of mRNA expression, which showed that HIF-1α-mediated transcription occupied the highest rank among pathways which are more active in glioblastomas compared to anaplastic astrocytomas (32). Since HIF-1(α) directly transactivates GLUT-1 and CA IX, our findings of concordant positivity and significantly correlated abundance of these proteins are consistent with theoretical considerations. Furthermore, the spatial distribution of the hypoxia-related proteins clearly showed a preference for the immediate vicinity of tissue necrosis, which is a well-known pattern described previously (15,33–35). This may suggest that necrosis, a pathognomonic sign of glioblastoma, is triggered by hypoxia; however, data supporting a role for alternative causative factors have also been reported [e.g., glucose depletion (36) and glutamate excitotoxicity (37)]. Differences in the quantitative expression levels of hypoxia-related markers in individual tumors, as observed in our study, are not surprising given the well-known fact that hypoxia-inducible proteins show substantial dissimilarities regarding magnitude (38) and the time course of induction by hypoxia as well as half-life upon re-oxygenation (39,40). Additionally, the influence of hypoxia may be modified by other variables of the tumor microenvironment [e.g., glucose availability (41)]. Similar variations between GLUT-1 and CA IX have been reported, e.g., in cancers of the uterine cervix (42).

A number of previous studies have already described an increase in the expression of the hypoxia-dependent proteins, HIF-1α, GLUT-1 and CA IX, with increasing malignancy grade [from grades II to IV) (11–13,17)]. However, this does not necessarily imply significant differences between grades III and IV, which were reported in only 2 of these studies (11,17). Surprisingly, a study by Flynn *et al*(16) not only showed a (non-significant) increase in GLUT-1 expression with tumor grade, but also reported a lower expression of CA IX in glioblastomas compared to anaplastic astrocytomas. Compared to our study, the prevalence of marker-positive cases in most of the aforementioned studies was far higher in terms of anaplastic astrocytomas (exceeding 50%) (11–13) and lower as regards glioblastomas (12,13,17). The following factors may be considered to explain these differences. First, marker expression in our study was analyzed in whole tumor sections, preventing false-negative results which may occur with smaller specimens due to the large heterogeneity of the expression of hypoxia-related markers. Second, assessment criteria of positivity of immunohistochemical staining were defined narrowly in our study. As described in the Materials and methods section, only a distinct, biologically justifiable staining at the level of individual cells was counted as a positive signal. Indeed, grade III tumors frequently exhibited cytoplasmic positivity for HIF-1α or GLUT-1. The intensity of this cytoplasmic signal, however, was on a far lower level compared to the specific signal which was observed (in the case of HIF-1α in tumor cell nuclei, in the case of GLUT-1 in the cell membrane of tumor cells, endothelial cells of autochthonous blood vessels of the brain and in erythrocytes). Third, we took methodological precautions to avoid artifacts that may arise from the false-positive staining of endogenous biotin. This source of error has been previously described as being relevant for rat brain tissues (43) and its relevance for human tissue has been documented by us in preliminary methodological experiments (data not shown). In the case of HIF-1α, which we detected with the extremely sensitive ‘catalyzed signal amplification’ system, we added additional steps to the protocol to actively block endogenous biotin. All other antigens were detected with a non-biotin-based detection system, for which this source of error does not play a role. The fact that our results quantitatively are both lower (as regards anaplastic astrocytomas) and higher (as regards glioblastomas) than other studies, argues against an overall inferior sensitivity of our immunohistochemical protocols.

As a second main result, we show that the proportion of tissue areas beyond diffusion distances of 80 and 120 μm (DLF_80_ and DLF_120_, respectively) is far greater in glioblastomas than in anaplastic astrocytomas. In keeping with this finding, the heterogeneity (i.e., standard deviation) of the mean ICDs in glioblastoma is also higher and GLUT-1- and CA IX-positive areas colocalize with areas of significantly higher mean diffusion distances. This substantial increase in diffusion distances (and possibly in hypoxic tissue areas) observed in our study, is consistent with the finding of an increase in the extent of EF5 binding in glioblastomas compared to anaplastic astrocytomas (8). Data on the quantification of the vasculature of glioblastoma are limited. Studies that have determined vascularization in so-called ‘hot spots’ (44), primarily investigate clinical utility as a prognostic parameter and may be compared only partially with the analysis described in our study. Quantitative approaches comparable to our study are found in the studies by Wesseling *et al*(45,46). These authors have also studied complete tumor sections of glioblastoma with the help of digital image analysis in comparison with normal brain white matter (46). In another study (45), astrocytomas of grade II to IV were compared to normal brain tissue. In both studies, it was shown that, similar to our results, glioblastomas exhibited a remarkably high standard deviation of all vascularization-associated parameters, which distinguished this entity from anaplastic astrocytomas and, in fact, all other entities examined. These studies also found an increased mean number, surface area, volume and diameter of the vessels examined in glioblastomas compared to lower grade gliomas and normal brain tissue. This observation does not contradict our data, since the parameters analyzed by us do not exclude the presence of a high number of vascular structures, but merely describe whether these structures are distributed in an uneven fashion, thereby leading to the occurrence of diffusion-limited areas. Furthermore, Yoshii and Sugiyama (28) showed decreased ICDs in gliomas (of different grades of malignancy) compared to normal white matter. Differences between their analysis and our study may primarily relate to a decisive difference in the sampling strategy. While our study excluded only macroscopic necrosis and performed an unbiased quantification on large tumor areas, Yoshii and Sugiyama investigated ICDs only in confined areas of high proliferation (as measured by bromodeoxyuridine staining). Additionally, Yoshii and Sugiyama (28) relied solely on visual identification of blood vessels in H&E sections, while our analysis is based on an endothelial-specific immunostaining. Finally, Evans *et al*(47) performed a study comprising 6 human brain tumors, among them 3 glioblastomas, using CD31 staining for microvessels and EF5 as a hypoxia marker. Contrary to our findings, these authors could not show uniform diffusion gradients surrounding individual microvessels. Alternatively, their data suggested that approximately equal numbers of tumor microvessels show positive and negative EF5 gradients. As negative gradients of hypoxia-related marker expression around individual blood vessels were not observed in the tumors analyzed in our study, we currently do not have an explanation for these diverging results.

Although the existence of a global spatial correlation between the expression of hypoxia-associated markers and diffusion distances in the present study was unequivocal, we observed local deviations from this pattern, particularly as regards the expression pattern of HIF-1α. Some tumor areas exhibited an expression of HIF-1α in a typical pattern, even though the local diffusion distances were low. It may be speculated that such areas could represent the morphological correlate of acute hypoxia, generated by the obstruction of tumor vessels due to microthrombosis, as described by Brat *et al*(20) or differences in the blood flow of feeding arterioles. Technical imperfections may also play a role in these mismatches in individual cases. While the registration of serial sections was possible with high precision, the use of 4-μm-thick specimens, as employed in the present study, may lead to distances along the z-axis which cannot be fully compensated by morphological operations. In a few specimens, optimal thresholding of CD34-positive pixels also proved to be more difficult, since the tumor cells also showed a signal.

The third essential finding arising from our data is the existence of a dual relationship between hypoxia and proliferative activity in glioblastoma. On the one hand, the intensity of proliferation was correlated with the magnitude of GLUT-1 expression on a global level. On the other hand, colocalization analyses revealed that microregional Ki67 tended to cluster outside of GLUT-1-positive areas. A global correlation between proliferation and GLUT-1 abundance, to our knowledge, has not been previously described in human glioblastoma, but has been reported for other tumor entities (48,49). Additionally, Zhong *et al*(33) described a correlation between higher proliferation and HIF-1α in 126 tumors of 13 entities. These data are commonly interpreted as being the result of a tumor, which exhibits a proliferation too high in relation to its angiogenic potency and this view is indeed compatible with the data of Szeto *et al*(21) (see Introduction). As inadequate feedback between endogenous and exogenous growth signals and the availability of oxygen may be responsible for the phenomenon of ‘outgrowing’ blood supply, it is of particular importance to consider that mTOR, an integration hub of both influences (22) is highly dysregulated in glioblastoma (24,25). Compatible with this suspected pathophysiology, GLUT-1-positive tumors also showed an increased mTOR activity in our study. However, residual responsiveness of mTOR to oxygen deprivation may be mirrored by our finding of a preferential aggregation of p-rpS6 outside of GLUT-1-positive areas. In selected cases this even corresponded to a nearly complementary pattern. While there is no doubt that mTOR can control proliferation *in vitro*, our *in vivo* finding of an at least partial decoupling of proliferation and mTOR in our colocalization analyses argues against a simple causal relationship between the dysregulation of mTOR and Ki67.

In conclusion, glioblastomas represent a tumor entity, which is shaped decisively by hypoxic tissue areas. Our results clearly show that the activation of hypoxia-induced protein expression is typically not observed in anaplastic astrocytomas. It therefore seems possible, even probable, that the minority of anaplastic astrocytomas which exhibit an activation of the hypoxic response biologically already represent the decompensated phenotype of glioblastoma. This is of great clinical interest, as it may represent a pathophysiological rationale for treatment of these patients, similar to glioblastoma.

## Figures and Tables

**Table I:** Antibodies (ABs), immunohistochemical technique and resulting staining patterns.

Antigen	Epitope retrieval buffer	Primary AB, Cat. no. dilution	Primary AB supplier	Detection system	Staining pattern
HIF-1α	Citrate pH 6.0	NB 100–123 mono^a^, 1:1000	Novus Biologicals, Littleton, CO, USA	CSA, Dako	Nuclear
GLUT-1	Citrate, pH 6.0	GI817C01 poly^b^, 1:200	DCS, Hamburg, Germany	ImmPRESS anti-rabbit vector	Membranous
CA IX	Citrate pH 6.0	ab15086 poly, 1:1000	Abcam, Cambridge, UK	ImmPRESS anti-rabbit vector	Membranous
CD34	Tris/EDTA pH 9.0	M7165 mono, 1:50	Dako, Hamburg, Germany	ImmPRESS anti-mouse vector	Membranous
Ki67	Tris/EDTA pH 9.0	ab16667 mono, 1:200	Abcam, Cambridge, UK	ImmPRESS anti-rabbit vector	Nuclear
p-rpS6	Citrate pH 6.0	#4857 mono, 1:75	Cell Signaling Technology Danvers, MA, USA	ImmPRESS anti-rabbit vector	Cytoplasmic

a Monoclonal antibody,

b polyclonal antibody.

**Table II:** Marker expression in glioblastoma vs. anaplastic astrocytoma.

% of tumor area	Glioblastoma		Anaplastic astrocytoma		Difference p-value
	Mean	Range	Mean	Range	
HIF-1α	1.3	0.1–4.9	0.1	0–0.9	0.001
GLUT-1	8.7	0.5–15.9	0.8	0–7.3	0.00004
CA IX	3.3	0.4–11.3	0.4	0–4.1	0.0004
Ki67	3.9	0.1–10.1	1.9	0.5–9.2	0.0986
p-rpS6	11.0	0.2–23.3	4.3	0.6–12.5	0.02416
